# Crystallization and Characterization of an Inflammatory Lectin Purified from the Seeds of *Dioclea wilsonii*

**DOI:** 10.3390/molecules16065087

**Published:** 2011-06-20

**Authors:** Thaiz Batista Azevedo Rangel, Ana Maria Sampaio Assreuy, Alana de Freitas Pires, Amanda Uliana de Carvalho, Raquel Guimarães Benevides, Rafael da Conceição Simões, Helton Colares da Silva, Maria Júlia Barbosa Bezerra, Antonia Samia Fernandes do Nascimento, Kyria Santiago do Nascimento, Celso Shiniti Nagano, Alexandre Holanda Sampaio, Plínio Delatorre, Bruno Anderson Matias da Rocha, Patricia Machado Bueno Fernandes, Benildo Sousa Cavada

**Affiliations:** 1 Núcleo de Biotecnologia, Centro de Ciências da Saúde, Universidade Federal do Espírito Santo, Vitória, ES 29040-090, Brazil; Email: thaizrangel@gmail.com (T.B.A.R); amandauliana@gmail.com (A.U.C.); 2 Instituto Superior de Ciências Biomédicas, Universidade Estadual do Ceará, P. O. Box 6043, 60455-970 Fortaleza, Ceará, Brazil; Email: anassreuy@gmail.com (A.M.S.A.); alana_pires@yahoo.com.br (A.F.P.); 3 Laboratório de Moléculas Biologicamente Ativas, Departamento de Bioquímica e Biologia Molecular, Universidade Federal do Ceará, P. O. Box 6043, 60455-970 Fortaleza, Ceará, Brazil; Email: raquelgb@gmail.com (R.G.B.); bio.rafael@gmail.com (R.C.S.); heltoncolares6@gmail.com (H.C.S.); mjuliabb@gmail.com (M.J.B.B.); asamiaf@yahoo.com.br (A.S.F.N.); kyriasantiago@ufc.br (K.S.N.); naganocs@gmail.com (C.S.N); sampaioa@ufc.br (A.H.S.); brunoanderson@gmail.com (B.A.M.R.); 4 Departamento de Biologia Molecular, Centro de Ciências Exatas e da Natureza - Campus I, Universidade Federal da Paraíba, Caixa Postal 5009, 58051-970, João Pessoa, PB, Brazil; Email: pldelatorre@gmail.com

**Keywords:** crystallization, *Dioclea wilsonii*, inflammation, lectin, tandem mass spectrometry

## Abstract

DwL, a lectin extracted from the seeds of *Dioclea wilsonii*, is a metalloprotein with strong agglutinating activity against rabbit and ABO erythrocytes, inhibited by glucose and mannose. DwL was purified by affinity chromatography on a Sephadex G-50 column and ion exchange chromatography on a HiTrap SP XL column. SDS-PAGE revealed three electrophoretic bands corresponding to the α (25,634 ± 2 Da), β (12,873 ± 2 Da) and γ (12,779 ± 2 Da) chains. Protein sequencing was done by Tandem Mass Spectrometry. The primary sequence featured 237 amino acids and was highly homologous to other reported Diocleinae lectins. A complete X-ray dataset was collected at 2.0 Å for X-Man-complexed DWL crystals produced by the vapor diffusion method. The crystals were orthorhombic and belonged to the space group I222, with the unit-cell parameters a = 59.6, b = 67.9 and c = 109.0 Å. DWL differed in potency from other ConA-like lectins and was found to induce neutrophil migration in rats, making it particularly useful in structural/functional studies of this class of proteins.

## 1. Introduction

Lectins are a class of proteins that specifically bind to carbohydrates and form complexes with molecules and biological structures containing saccharides, without altering the covalent structure of the glycosyl ligands [1,2]. Furthermore, lectins play an important role in many cellular processes by deciphering the glycocodes encoded in the structure of glycans attached to soluble and integral cell-membrane glycoconjugates. These processes include cell communication, host defense, fertilization, development, parasitic infection and tumor metastasis [3]. Lectins are ubiquitously distributed in plants, animals and microorganisms. However, most research efforts have been focused on plant lectins, especially from the Leguminosae family. In this family, lectins make up as much as 10% of the total nitrogen in mature seed extracts [4]. 

Legume lectins constitute a highly homologous family of proteins. Homology is greater among lectins from the same family [5]. Lectins from the genera Canavalia, Cratylia and Dioclea make up a group of well-conserved glucose/mannose-binding lectins with many chemical and physicochemical properties in common. These proteins are formed by post-translational circular permutation, cleaving the pre-pro-protein in two small chains (β and γ). The active α chain (α = γ + β) is circularly permuted in primary sequence relative to its own inactive γ-β precursor [6]. This group includes ConA, extracted from the seeds of *Canavalia ensiformis*, the most extensively studied of all lectins.

Despite their great amino acid sequence homology, Diocleinae lectins have been shown to differ remarkably with regard to biological activity. Thus, several lectins in this group are actually more potent than ConA [7]. The biological activities that have been investigated for Diocleinae lectins include mitogenic stimulation and γ-interferon production in human lymphocytes [8], histamine release [9], induction of edema and peritonitis [10], macrophage stimulation and leukocyte accumulation [11]. The range and effectiveness of biological responses elicited by Diocleinae lectins make them valuable tools in many areas of medicine and suggest a potential for future use as therapeutic agents. Furthermore, lectins in this group are excellent models for the study of relation between minor structural differences and functional properties [12]. 

*Dioclea wilsonii*, commonly known as bull’s eye, belongs to the class Liana (woody vines), a major structural component of the Brazilian Atlantic forest. The species is still found in ecological reserves protected from long-standing deforestation [13]. However, many biotechnically promising plant species endemic to this area are currently endangered and may become extinct without being characterized. The objective of this study was to extract the lectin DwL from the seeds *Dioclea wilsonii*, solve its primary and secondary structures and evaluate its effect on acute inflammation using an *in vivo* rat peritonitis model. 

## 2. Results and Discussion

Hemagglutinating activity (HA) against rabbit and ABO human erythrocytes was observed for the soluble protein extracted from the seeds of *D. wilsonii*. This activity was used to determine the active chromatographic fractions required to purify DwL. The addition of trypsin and papain greatly increased HA and the highest levels of HA (131,072 HU/mL) were observed for rabbit erythrocytes treated with trypsin. Presumably, carbohydrates that compose erythrocyte glycans are more exposed after proteolytic digestion.

Furthermore, DwL displayed affinity for simple sugars such as D-glucose and D-mannose at minimal inhibitory concentration of 62.5 mM, confirmed findings from studies on others lectins of the genus Dioclea, such as *D. altissima* [14], *D. rostrata* (DRL) [15], *D. violacea* (DVL) [16], *D. virgata* (Dvir) [17], *D. guianensis* (Dgui) [18] and *D. grandiflora* (DGL) [19]. This carbohydrate-binding specificity is believed to be a common feature of all Diocleinae lectins [20,21].

**Figure 1:**
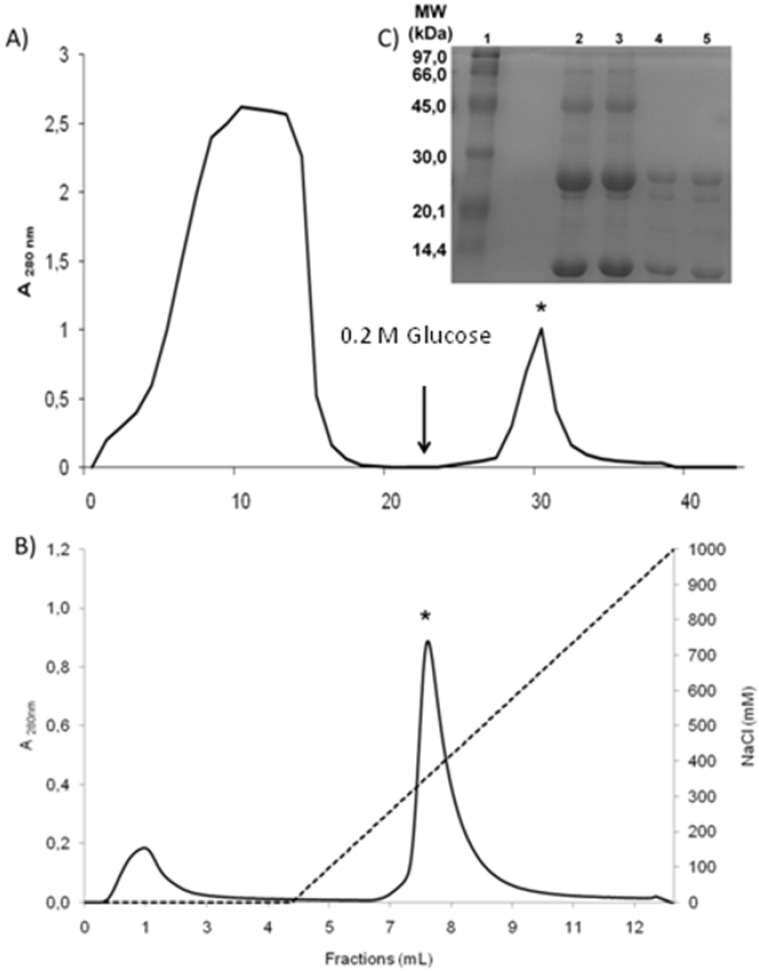
Purification of lectin from the seeds of *Dioclea wilsonii* (DwL). (**a**) Partial purification of DwL by affinity chromatography on a Sephadex G-50 column. The column was equilibrated and eluted with 0.1 M Tris-HCl buffer (pH 7.4) containing 0.15 M NaCl to remove the unbound proteins. Lectin (*Peak II) was recovered with 0.2 M Glucose or with 0.1 M Glycine buffer (pH 2.6) containing 0.15 M NaCl. (**b**) Purification of DwL by cation exchange chromatography on a SP Sepharose™ XL column using peak II from the affinity chromatography step. The column was equilibrated and eluted by a saline gradient (0–1 M NaCl) to remove contaminants (Peak I). Lectin (*Peak II) was recovered with Acetate buffer (pH 4.5). (**c**) SDS-polyacrylamide gel electrophoresis (15%) of DwL in the presence of β-mercaptoethanol. Lane 1: molecular mass markers [phophorylase b (97 kDa), albumin (66 kDa), ovalbumin (45 kDa), carbonic anhydrase (30 kDa), trypsin inhibitor (20.1 kDa) and α-lactalbumin (14.4 kDa)]. Lanes 2 and 3: Peak II of the Sephadex G-50 column. Lanes 4 and 5: Peak II of the SP Sepharose™ XL column.

DwL was easily purified in two steps by Sephadex G-50, a dextran affinity chromatography (Figure 1a) followed by ion exchange chromatography (Figure 1b). The purification efficiency is reported in Table 1. 

**Table 1:** Overall protein recovery and haemagglutinating activity of *Dioclea wilsonii* seeds at various stages of purification.

Fractions *	V (mL)	Protein			Hemagglutinating activity †			Minimum Concentration(mg/mL)	Purification
		mg/mL	Total	%	U.H./mL	Total	Specific		
Total Extract	30	145.62	4368.6	100	131072	3932160	900.09	1.11 × 10^−3^	1
Sephadex G-50 PII	14	4.26	59.64	1.36	32768	458752	7692.02	1.3 × 10^−4^	8.5
HiTrap SP XL PII	2	1.127	2.254	0.77	65536	131072	50150.85	1.3 × 10^−5^	55.72

* All fractions were recovered after dialysis and freeze drying and tested in rabbit erythrocytes; **^†^** Data obtained with trypsin treated erythrocytes.

**Figure 2:**
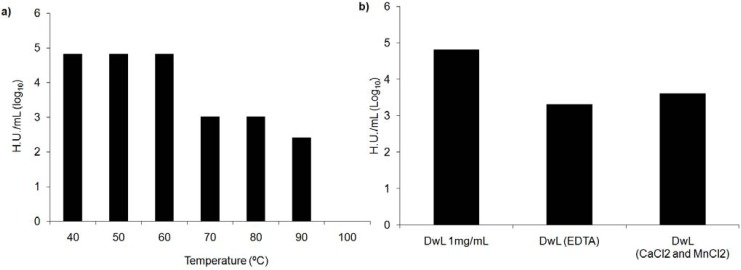
Stability of *Dioclea wilsonii* (DwL) hemagglutinating activity. (**a**) DwL thermostability. (**b**) DwL metal dependence. HA against trypsinized rabbit erythrocytes (0.2 M ultrapure EDTA followed by 0.15 M ultrapure NaCl) in presence or not of CaCl_2_ and MnCl_2_.

DwL was thermostable up to 60 °C. HA waned between 60–80 °C, becoming completely inactive at 90–100 °C (Figure 2a). HA against trypsinized rabbit erythrocytes was slightly inhibited by dialysis of the native protein with 0.2 M ultrapure EDTA followed by 0.15 M ultrapure NaCl.

However, the addition of Ca^+2^ and Mn^+2^ (5 mM) partly recovered lectin activity (Figure 2b). The fact that HA is inhibited by EDTA chelanting agents and recovered by the addition of metal ions indicates Diocleinae lectins in general are metal-dependent [14,17]. However, inhibition was very slight in the case of DwL, suggesting it may be a metalloprotein.

**Figure 3:**
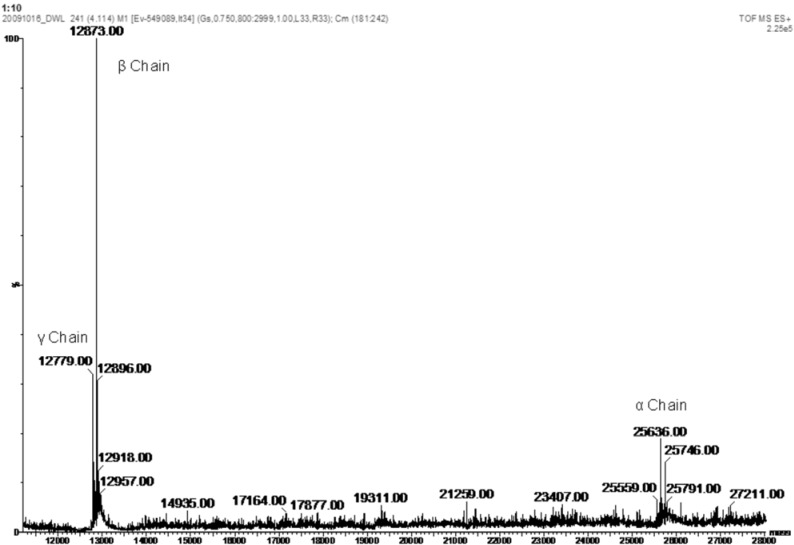
Deconvoluted mass spectra of lectin from the seeds of *Dioclea wilsonii* (DwL) acquired by ESI ionization in a hybrid quadrupole/ion mobility separator/orthogonal acceleration time-of-flight mass spectrometer.

The apparent molecular weight of DwL (25 kDa) and fragments (β and γ – 12 kDa) was estimated by SDS-PAGE in the presence of β-mercaptoethanol (Figure 1c). The electrophoretic profile of DwL was similar to that of other Diocleinae lectins, such as ConA (*Canavalia ensiformis*) [22], DGL (*Dioclea grandiflora*) [19], ConBr (*Canavalia brasiliensis*) [23], CFL (*Cratylia floribunda*) [24], Dgui (*Dioclea guianensis*) [18], ConM (*Canavalia maritima*) and DLLI (*Dioclea lehmanni*) [25]. In fact, it is well established that Diocleinae lectins are composed of one intact subunit (α) and two fragments (β and γ) originating from post-translational processing of the lectin precursor during seed development [26]. Thus, like ConA, DwL is composed of three chains (α, β and γ) and is expressed as a pre-pro-protein (_Nterm_signal peptide + γ chain + linker peptide + β chain + _Cterm_signal peptide) cleaved into β and γ products. Thus, the active protein (α chain) is formed by the fusion of the two smaller chains (β and γ) in inverse order, without the signal peptides and the linker peptide [6,26]. These findings were confirmed by mass spectrometry analysis proving the existence of just one lectin, composed of three chains (α, β and γ). The α-chain has a molecular mass of 25,634 ± 2 Da. The mass of each fragment is 12,873 ± 2 Da (β-chain) and 12,779 ± 2 Da (γ-chain) (Figure 3). The final sequence of DwL, as determined by tandem mass spectrometry, features 237 amino acids, distributed between the β-chain (residues 1–118) and the γ-chain (residues 119–237). The entire protein has a calculated molecular weight of 25.63 kDa and a theoretical pI of 5.3.

**Figure 4:**
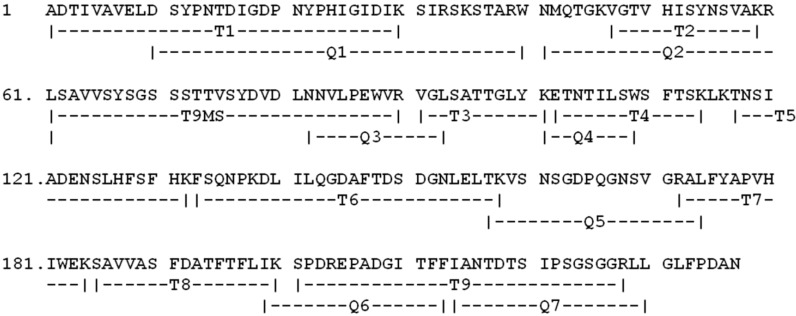
Complete amino acid sequence of lectin from the seeds of *Dioclea wilsonii* (DwL). Peptide fragments obtained by degradation of DwL with the endoproteinases trypsin (T) and chymotrypsin (C).

Figure 4 shows the complete sequence and Table 2 lists all sequenced peptides and their respective molecular masses. 

**Table 2:** Sequenced peptides of *D. wilsonii* lectin (DwL) and their respective molecular masses.

Peptide	Experimental Mass (Da)	Sequence
T1	3254.5566	ADTIVAVELDSYPNTDIGDPNYPHIGIDIK
T2	1373.7165	VGTVHISYNSVAK
T3	1108.5643	VGLSATTGLYK
T4	1512.7244	ETNTILSWSFTSK
T5	1845.8464	TNSIADENSLHFSFHK
T6	2865.4045	FSQNPKDLILQGDAFTDSDGNLELTK
T7	1472.7366	ALFYAPVHIWEK
T8	1715.9243	SAVVASFDATFTFLIK
T9MS	2864.2444	SPDREPADGITFFIANTDTSIPSGSGGR
Q1	3515.7151	DSYPNTDIGDPNYPHIGIDIKSIRSKSTARW
Q2	2303.1687	MNQTGKVGTVHISYNSVAKRL
Q3	1394.7444	NNVLPEWVRVGL
Q4	1090.5643	KETNTILSW
Q5	1785.8522	TKVSNSGDPQGNSVGRAL
Q6	1544.7365	IKSPDREPADGITF
Q7	1691.8844	FIANTDTSIPSGSGGRL

**Figure 5:**
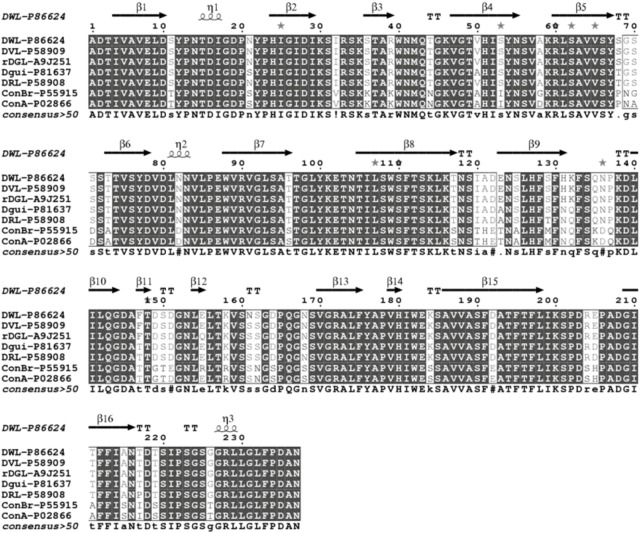
Primary sequence analysis. (a) Multiple alignment of rDGL, DVL, Dgui and DRL shows the highly conserved sequence in the secondary structure.

The full sequence of DwL is deposited in the Uniprot database under code number P86624. Comparisons with other sequence from the NCBI data bank revealed that the DwL is highly homologous with other Diocleinae lectins, especially those of the genus Dioclea. The lectins most similar to DwL are rDGL: 99% (SwissProt acession code: A9J251), DVL: 99% (SwissProt accession code: P58909), DRL: 96% (SwissProt accession code: P58908) and Dgui: 95% (SwissProt accession code: P81637). Multiple alignments of DwL, rDGL, DVL, DRL and Dgui are presented in Figure 5. 

DwL differs from rDGL in two amino acids at positions 155 and 161, and differs from DRL in eight amino acids at positions 68, 123, 129, 131, 132, 147, 161 and 217. The highly conserved carbohydrate and metal-binding sites in DwL contain the same residues described for other ConA-like lectins [27]. The protein secondary structures predicted with bioinformatics (PSSP: Protein Secondary Structure Prediction) provides a pointer towards determining a protein’s tertiary structure, which may be used to identify and classify protein domains into families. It may not be an accurate representation of the actual structure of a protein, but it is a good starting point for predicting binding pockets and protein-protein interaction sites. Figure 5 shows a representative sequenced peptide containing a carbohydrate-binding loop sequence.

**Figure 6:**
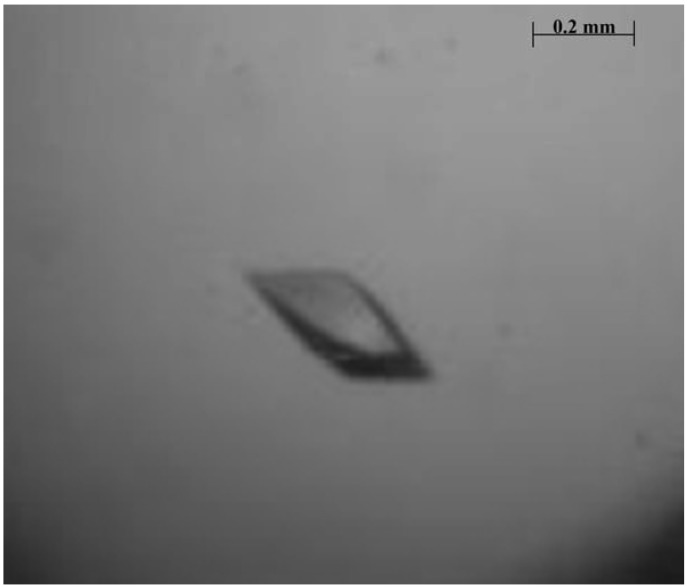
Crystal of lectin from the seeds of *Dioclea wilsonii* complexed with X-Man (5-Bromo-4-chloro-3-indolyl-α-D-mannopiranoside). The crystal belongs to orthorhombic space group I222.

Some crystals were obtained using crystallization condition number 41 from the Crystal Screen II kit (0.01 M Nickel (II) chloride hexahydrate, 0.1 M Tris-HCl pH 8.5, 1.0 M Lithium sulfate monohydrate). Optimizations were made for this condition. The best condition was 0.01 M Nickel (II) chloride hexahydrate, 0.1 M Tris-HCl pH 8.0, 1.0 M Lithium sulfate monohydrate. Crystals of a suitable size for X-ray diffraction analysis were obtained only in the presence of X-Man (Figure 6). The crystals obtained belonged to orthorhombic point group I222, with the cell parameters a= 59.65, b= 67.97, c= 109.04 Å, α = β = γ = 90°. Based on a molecular weight of 25,634 ± 2 Da, the Matthews coefficient (2.17 Å^3^ Da^−1^) [28] indicated the crystal contained 43.28% solvent. The asymmetric unit was found to be a monomer. The data collection statistics are shown in Table 3.

**Table 3:** Refinement statistics for crystal structure data collection of the lectin from the seeds of *Dioclea wilsonii* (DwL).

Data Collection *	
Point group	I222
Unit cell parameters (Å)	
A	59.6
B	67.9
C	109.0
Unit cell angles (°)	
α = β = γ	90
Total number of reflection	50274 (6863)
Total number of unique observations	10483 (1526)
Resolution limit (Å)	23.29–2.2
R_merge_ (%) ^a^	8.4 (24.1)
Completeness (%)	97.1 (98.5)
Multiplicity (%)	4.8 (4.5)
(I)/σ	5.0 (1.8)
Matthews coefficient (Å^3^.Da^−1^)	2.17
Solvent content (%)	43.28
Wavelenght (Å)	1.42

* Values in parenthesis represent the high resolution shell; ^a^ 
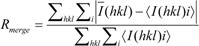
 where *I(hkl)i* is the intensity of i^th^ measurement of the reflection h and *I(hkl)* is the mean value of the *I(hkl)i* for all I measurements.

**Figure 7:**
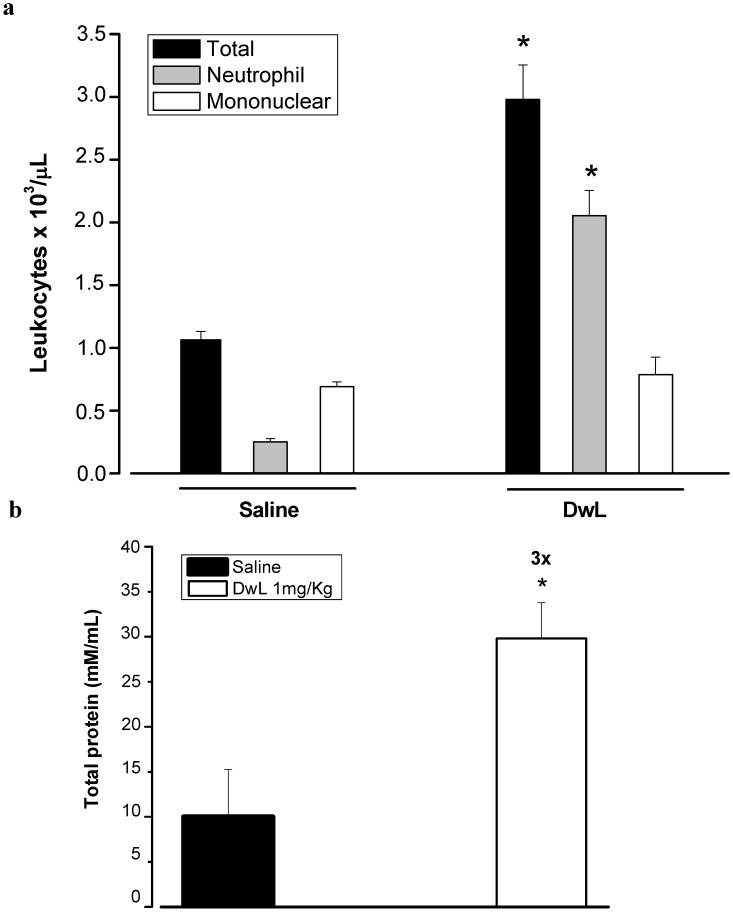
Neutrophil migration induced by lectin from the seeds of *Dioclea wilsonii* (DwL). DwL (1 mg/Kg) was administered intraperitoneally. Five hours later, the exudates were collected by washing with 10 mL saline containing 5 UI heparin/mL. (**a**) Total and differential cell counts were performed. (**b**) Total protein was quantified (A = 595 nm). Results are mean values ± S.E.M. for each group of 6 animals. * p < 0.05 compared to saline (ANOVA-Bonferroni).

Several studies have demonstrated the ability of plant lectins to activate cells of the immune system by different mechanisms. Lectins have thus become important tools for studying inflammatory cellular events and a relationship between lectin structures and biological effects has been proposed [29,30,31,32,33]. In this context, we investigated the *in vivo* effect of DwL on leukocyte migration, an important inflammatory cellular event. Intraperitoneal administration of DwL in rats caused significant leukocyte migration, mainly due to neutrophil migration (2053.21 ± 200.64 cell/ µL) when compared to the saline group (250.92 ± 27.27 cells/µL) (Figure 7). 

DwL increased the number of neutrophils in rat peritoneal cavities, without altering the number of mononuclear cells, a typical sign of acute inflammation. Other Diocleinae lectins, such as DRL [34], DGL, ConBr and ConA [10] have also been shown to elicit inflammatory responses. The ability of DwL to induce leukocyte migration was similar to that of ConA but inferior to that of DRL [34], DGL and ConBr [10]. In addition, DwL induced three times more vascular permeability than saline (30.09 ± 2 *versus* 10.1 ± 2.6 mM/mL). Similar effects have been described for other lectins [29,35].

## 3. Experimental

### 3.1. Materials

Seeds were collected from wild *D. wilsonii* plants located in Sooretama, a small town in the state of Espírito Santo, Southeastern Brazil. Rabbit erythrocytes were obtained from the Universidade Federal do Ceará (UFC). Blood from healthy human donors was provided by a public blood bank in Espírito Santo. The reagents were purchased from Sigma™ and GE Healthcare™.

### 3.2. Protein Extraction Procedure

The seeds were ground to a fine powder using a coffee mill and the soluble proteins were extracted at room temperature in 0.1 M Tris-HCl buffer pH 7.4 contining 0.15 M NaCl [1:10 (w:v)] under continuous stirring for 4 hours, followed by filtration through filter paper (Whatman™) and centrifugation at 10,000 × g at 4 °C for 20 min. The supernatant was used for further experiments. The protein concentration was determined by the method described by Bradford [36], using bovine serum albumin (BSA) as standard.

### 3.3. Hemagglutinating Activity

HA was determined in tubes by double serial dilutions. All tubes received 100 μL 0.1 M Tris–HCl buffer pH 7.6 containing 0.15 M NaCl. A 100 μL aliquot of supernatant was added to the first tube of the series. Subsequently, 100 μL suspension of 2% rabbit or ABO human erythrocytes suspension treated with trypsin, papain or neither, containing 0.15 M NaCl, was added to each tube. HA was measured after 30 min of incubation at 37 °C and 30 min of incubation at room temperature. HA was expressed as a titer (the reciprocal value of the highest dilution testing positive) per mg of protein.

### 3.4. Sugar Specificity

The sugar specificity was determined by comparing the ability of two sugars, D-glucose and D-mannose, to inhibit HA. DwL was incubated with the sugars at 37 °C for 30 min prior to determination of the inhibition titer, following the methodology described in section 3.3. The initial concentration of the carbohydrates tested was 0.5 M. Results were expressed as the minimum concentration of sugar required to inhibit HA.

### 3.5. Protein Purification

The supernatant obtained during protein extraction was applied onto a Sephadex G-50 (a cross-linked dextran – 30 mL) column previously equilibrated with 0.1 M Tris-HCl buffer (pH 7.4) containing 0.15 M NaCl. The unbound material was washed with the same buffer at a constant flow rate until the absorbance of the effluent at 280 nm had been stabilized at 0.05. The retained fraction was eluted with 0.2 M glucose or 0.1 M Glycine buffer (pH 2.6) containing 0.15 M NaCl, until the absorbance of the effluent at 280 nm had been stabilized at 0.05, and dialyzed exhaustively for 12 h at 5 °C against ultrapure water. The retained fraction was freeze-dried and further purified by high performance liquid chromatography (ÄKTA purifier 10/100, Amersham Pharmacia Biotech, England) on a SP Sepharose™ XL (strong cation) column using a saline linear gradient (0–1 M NaCl) containing acetate buffer (pH 4.5). DwL was extensively dialyzed against ultrapure water and lyophilized for further analysis, including thermostability, effect of EDTA on DwL activity, molecular mass determination by mass spectrometry and crystallization trials. 

### 3.6. Sodium Dodecyl Sulfate Polyacrylamide Gel Electrophoresis

Following the method of Laemmli [37], sodium dodecyl sulfate (SDS) polyacrylamide gel electrophoresis (PAGE) was carried out in 0.75 mm vertical gel slabs at of a 15% polyacrylamide separation gel containing 1.5 M Tris–HCl (pH 8.8), 30% acrylamide/0.8% bis-acrylamide, 10% ammonium persulfate, tetramethylethylenediamine (TEMED) (pH 8.9) and 10% SDS buffer, and 4% stacking gel containg 0.5 M Tris–HCl (pH 6.8), 30% acrylamide/0.8% bis-acrylamide, 10% ammonium persulfate, tetramethylethylenediamine (TEMED) (pH 8.9) and 10% SDS buffer. Samples were dissolved in 0.88 M Tris–HCl (pH 6.8), 2% SDS buffer, 5% β-mercaptoethanol, 1% bromophenol blue and 12.5% glycerol, followed by incubation at 100 °C for 5 min. Electrophoresis was conducted at a constant current of 25 mA for 90 min. The protein bands were visualized by staining with Coomassie Brilliant Blue R-250 [38]. The molecular markers were phosphorylase b (97kDa), albumin (66kDa), ovalbumin (45kDa), carbonic anhydrase (30kDa), trypsin inhibitor (20.1kDa) and α-lactalbumin (14.4kDa). 

### 3.7. Lectin Metal Dependence

The metal dependence of the purified lectin (1 mg pure DwL dissolved in 1 mL pure water) was determined after 48 hours of dialysis against 0.2 M ultrapure EDTA, followed by dialysis against 0.15 M ultrapure NaCl for 24 hours. The dialyzed lectin solution was evaluated for HA. Recovery of HA was done by adding 5 mM CaCl_2_ and 5 mM MnCl_2_. 

### 3.8. Lectin Thermostability

The effect of temperature on DwL activity was investigated by incubation of the lectin solution (1 mg DwL/mL) at different temperatures (30; 40; 50; 60; 70; 80; 90 and 100 °C) for 1 hour, followed by evaluation of HA. 

### 3.9. MW Determination by Mass Spectrometry

The molecular mass of DwL was determined by electrospray ionization using a hybrid mass spectrometer (the Synapt HDMS system-Waters Corp., Milford, USA). A protein solution (10 ρmol/mL) was infused into the system at a flow rate of 10 mL/min. The capillary voltage and the cone voltage were set at 3 kV and 40 V, respectively. The source temperature was maintained at 473 K and nitrogen was used as a drying gas (flow rate of 150 L/h). The data were acquired with the software Mass Lynx 4.0. The multiply charged spectra were deconvoluted using maximum entropy techniques [39].

### 3.10. Protein Digestion and Tandem Mass Spectrometry Analysis

The SDS-PAGE bands were excised and bleached in a 50 mM ammonium bicarbonate solution in 50% acetonitrile. The bands were dehydrated in 100% acetonitrile and dried in a speedvac (LabConco). The gel was rehydrated with a 50 mM ammonium bicarbonate solution containing trypsin (Promega) or chymotrypsin (Sigma) (1:50 w/w; enzyme: substrate ratio) at 310 K overnight. Peptides were extracted from the gel, concentrated and injected into a Nanoacquity system connected to the electrospray source of a mass spectrometer (SYNAPT HDMS system-Waters Corp., Milford, USA). The sample was applied to a C18 chromatography column (75 µm × 100 mm) and eluted with a 10–85% acetonitrile gradient containing 0.1% formic acid. The mass spectrometer operated in positive mode, using a source temperature of 363 K and capillary voltage of 3.0 kV. The LC-MS/MS experiment was performed with the DDA (data-dependent acquisition) function selecting for MS/MS experiments with doubly or triply charged precursor ions fragmented by collision-induced dissociation. The data were processed and analyzed with a Proteinlynx v2.4 (Waters) using the peptide mass fingerprint (PMF) and the peptide fragmentation pattern as search parameters. Some peptide sequences were obtained by *de novo* sequencing. 

### 3.11. Primary Structure Analysis

The primary sequence was submitted to BLAST [40]. The proteins with the best e-value were selected for Multalin alignment [41]. The alignment with secondary structure prediction was made with the software ESPript 2.2 [42]. The theoretical Mw and pI were calculated with the software ProtParam [43].

### 3.12. Crystallization and Data Collection

DwL crystals were grown with the hanging-drop vapor diffusion method at room temperature (293 K). The protein was dissolved at 10mg/mL in ultrapure water and incubated for one hour with 3 mM X-Man (5-bromo-4-chloro-3-indolyl-a-D-mannopyranoside). X-Man was dissolved at 30 mM in pure DMSO. X-Man contains mannose which helps stabilize the binding site and seems to help in crystal packing. The sparse matrix method for Crystal Screen I and II (Hampton Research™) was used to determine the best conditions for crystallization [44]. Drops were prepared by mixing 2.0 µL from the protein solution and 2.0 µL reservoir solution. The reservoir was filled with 300 µL of crystallization condition. X-ray diffraction data were collected at a maximum resolution of 2.0 Å, cooled to 100 K. To avoid ice formation, 30% glycerol was used as a cryoprotectant. The X-ray diffraction data were collected at 1.42 Å wavelength at a beamline MX1 station (National Laboratory of Synchrotron Light-LNLS, Campinas, Brasil) using a CCD (MAR research) detector placed 100 mm from the crystal. A set of 120 frames with an oscillation range of 1° was recorded. Diffraction data were indexed, integrated and scaled using the software iMOSFLM and SCALA [45,46]. 

### 3.13. Biological Assays

#### 3.13.1. Animals, Drugs and Reagents

Wistar rats (180–250 g) were kept in cages (6 in each) in a controlled environment (circadian cycle, 25 °C, food and water *ad libitum*). The experimental protocols were approved by the Institutional Animal Care and Use Committee of the State University of Ceará (UECE, Fortaleza, Ceará, Brasil) under No. 10130208-8/40, following the recommendation of the Guide for the Care and Use of Laboratory Animals of the US Department of Health and Human Services (NIH publication No. 85–23, revised 1985).

#### 3.13.2. Peritonitis Model

DwL (1 mg/Kg) or saline was administered intraperitoneally. Five hours later the animals were euthanized. Cells were harvested by washing the peritoneal cavities with 10 mL saline (5 IU heparin) for total and differential leukocyte counts (neutrophils, eosinophils, mast cells and mononuclear cells) (Cytopro 7620, Wescor) [47]. Results were expressed as cells/µL of peritoneal wash. The increase in vascular permeability was quantified by extravasation of plasma protein using the Bradford Method [36].

#### 3.13.3. Statistical Analysis

Results were presented as mean values ± SEM for each group of 6 animals. Differences were analyzed with one-way ANOVA followed by the Bonferroni test. The levels of statistical significance were set at *p* < 0.05.

## 4. Conclusions

DwL is a glucose/mannose-binding lectin purified from the protein extract of the seeds of *Dioclea wilsonii*. It is a monomer with molecular mass of 25,634 ± 2 Da that associates as a tetramer. DwL is synthesized as a single product (α-chain) through post-translational circular permutation, cleaving the pre-pro-protein in two small chains (β and γ). The α-chain polypeptide has 237 amino acids and is classified as a Con-A like lectin based on its primary amino acid sequence. DwL, complexed to X-man, was crystallized in order to determine its structure by X-ray crystallography and to better understand its carbohydrate recognition and dimer/tetramer association. X-Man complexed with DwL enhanced crystallization and permitted a more accurate X-ray data diffraction analysis. Detailed three-dimensional mapping of the structure of DwL, including the architecture of the X-Man molecule, is under refinement and the complete DwL crystal structure is currently being resolved in our laboratory. The present study also demonstrated that DwL elicits an acute inflammatory response by induction neutrophil migration. This important finding, in combination with a clearer picture of the tertiary structure of DwL, may help clarify the structural/functional relation between this protein and its ligands.
